# Differences in Visuo-Motor Control in Skilled vs. Novice Martial Arts Athletes during Sustained and Transient Attention Tasks: A Motor-Related Cortical Potential Study

**DOI:** 10.1371/journal.pone.0091112

**Published:** 2014-03-12

**Authors:** Javier Sanchez-Lopez, Thalia Fernandez, Juan Silva-Pereyra, Juan A. Martinez Mesa, Francesco Di Russo

**Affiliations:** 1 Departamento de Neurobiología Conductual y Cognitiva del Instituto de Neurobiología, Universidad Nacional Autónoma de México, Juriquilla, Querétaro, México; 2 Proyecto en Neurociencias de la Facultad de Estudios Superiores Iztacala, Universidad Nacional Autónoma de México, Tlalnepantla, Estado de México, México; 3 Departamento de Psicología del Deporte, Instituto de Medicina del Deporte de Cuba, Cerro, Ciudad de La Habana, Cuba; 4 Dipartimento di Scienze Motorie Umane e della Salute, Università degli Studi di Roma “Foro Italico,” Roma, Lazio, Italia; 5 Unità di Neuropsicologia, IRCCS Fondazione Santa Lucia, Roma, Lazio, Italia; University of Texas at Dallas, United States of America

## Abstract

Cognitive and motor processes are essential for optimal athletic performance. Individuals trained in different skills and sports may have specialized cognitive abilities and motor strategies related to the characteristics of the activity and the effects of training and expertise. Most studies have investigated differences in motor-related cortical potential (MRCP) during self-paced tasks in athletes but not in stimulus-related tasks. The aim of the present study was to identify the differences in performance and MRCP between skilled and novice martial arts athletes during two different types of tasks: a sustained attention task and a transient attention task. Behavioral and electrophysiological data from twenty-two martial arts athletes were obtained while they performed a continuous performance task (CPT) to measure sustained attention and a cued continuous performance task (c-CPT) to measure transient attention. MRCP components were analyzed and compared between groups. Electrophysiological data in the CPT task indicated larger prefrontal positive activity and greater posterior negativity distribution prior to a motor response in the skilled athletes, while novices showed a significantly larger response-related P3 after a motor response in centro-parietal areas. A different effect occurred in the c-CPT task in which the novice athletes showed strong prefrontal positive activity before a motor response and a large response-related P3, while in skilled athletes, the prefrontal activity was absent. We propose that during the CPT, skilled athletes were able to allocate two different but related processes simultaneously according to CPT demand, which requires controlled attention and controlled motor responses. On the other hand, in the c-CPT, skilled athletes showed better cue facilitation, which permitted a major economy of resources and “automatic” or less controlled responses to relevant stimuli. In conclusion, the present data suggest that motor expertise enhances neural flexibility and allows better adaptation of cognitive control to the requested task.

## Introduction

Sports performance depends on many different components, including cognitive and motor skills. Previous studies have reported the superior cognitive abilities of expert athletes, who are capable of quickly extracting important information and using this ability to identify the most relevant information [1], [2], [3], [4], [5]. In addition, experts can better modulate their cognitive and motor resources according to specific task demands [6].

A method that is commonly used to study how sports performance is enhanced is the analysis of the brain electrical activity. Previous studies have reported differences between expert athletes, less expert athletes and/or non-athletes studying EEG rhythms during eyes-closed resting state and concluding that these rhythms are enhanced in elite athletes compared to control subjects [7]. Additionally, this research group has investigated the task-related power decrease of EEG alpha activity as an index of cortical activation and they have observed that athletes' brains is characterized by a reduced cortical activation during a motor task [8], [9], a sport judgments task [10] and the reactivity to eyes opening in the condition of resting state [11], in line with the “neural efficiency” hypothesis. Another analysis commonly used has been the event-related potentials (ERPs), which are regarded as direct correlates of information processing [12], [13]. Most of the previous ERP studies on athletes have compared experts with beginners or non-athletes and have found differences between groups in the amplitude and latency of different ERP components. These differences have been associated with expertise, sports discipline and level of task demand [14], [15], [16], [17], [18], [19], [20], [21], [22]. Additionally, movement-related cortical potential (MRCP) has been studied in this population. This activity has been associated with motor skill learning and performance. During a self-paced task, in the final seconds prior to voluntary movement production, there is an increase in electrical activity in the premotor and motor areas of the brain. One component of the MRCP, the Bereitschafts potential (BP), is a slowly rising negativity that occurs 1–2 s prior to movement onset. The BP is followed by a steeper gradient negativity, the negative slope (NS'), 400–500 ms prior to movement onset. These components are followed by the motor potential (MP), with the peak negativity occurring concomitantly with movement onset in contralateral central sites [23].

Several studies have investigated differences in the amplitude and onset time of the MRCP between expert and novice performers to aid our understanding of learning-related changes in brain functioning [24], [25], [26], [27], [28], [29]. The main findings from these studies are that expert performers show smaller amplitude and later onset MRCPs prior to task performance than their novice counterparts. This fact has been observed in groups of expert clay target [24] and pistol shooters [30], elite and novice kendo martial art performers [25], [26] and guitar players [27], [28], [29]. It has been concluded that experienced performers are able to plan and perform the task with reduced cortical activity compared to novices and that these differences can be attributed to long-term training by the expert group.

Compared to the aforementioned MRCP studies for self-paced movements, the MRCP in a stimulus-related task (externally triggered movements) has not been sufficiently studied in general and has never been studied as a function of expertise. Therefore, features of this brain electrical activity have not been defined as a function of expertise during stimulus-related tasks. In this sense, differences in MRCP between expert and novice athletes could be related not just to expertise but also to cognitive task demand, as found in ERP studies. Externally triggered MRCPs differ from self-paced MRCPs because external events affect motor preparation. Starting from stimulus onset, MRCP negativity is followed by a strong positivity in prefrontal and parietal areas, identified as the P3 complex in ERP studies. This activity depends on stimulus discrimination, response selection and execution. While parietal activity has been extensively studied because it corresponds to components of the ERP [31], prefrontal activity was discovered only recently and has been related to aging [31] and physical exercise [32]. The main components of this prefrontal activity are referred to as prefrontal negativity [33] [14] and prefrontal positivity (pP), depending on polarity, with pN preceding pP.

Many cognitive processes are required during athletic training and competition, including different types of attention, such as sustained attention, which maintains an athlete's focus throughout the competition, and transient attention, which allows perception of an opponent's cues, enabling a fast response. However, in the sport psychology literature, these processes have not been clearly defined. Previous ERP studies have shown that sustained and transient attention are processes clearly separated [34]. Moreover, skilled and novice athletes exhibit differences in both types of attention. Given that MRCP has been associated with motor skill learning and performance and also that externally triggered tasks involve an interaction between MRCP and cognitive processes, we hypothesized that two different processes would be found and that they would be reflected by differences in behavioral performance and MRCP between groups. Skilled athletes must show better capabilities in both sustained and transient attention tasks than novices. If skilled athletes are given a highly demanding task (i.e., physical or cognitive), they will use all of the necessary resources required by the task, whereas if an easier task is presented, they will be economical and will use less resources in responding to the task. Novice athletes have not developed all of the necessary resources to control their responses during a high-demand task. The goal of the present study was to identify behavioral and electrophysiological differences in MRCP between skilled and novice martial arts athletes during two different types of tasks: a sustained attention task and a transient attention task.

## Experiment 1

To investigate the differences in MRCP between skilled and novice martial arts athletes on a sustained attention task, a continuous performance task (CPT) was used. In sustained attention tasks, participants are instructed in advance to attend to the same specific stimulus across all stimuli presented in the task. Every stimulus is a potential target that may require a response [34]. This task requires high levels of sustained attention. Given that combat requires long periods of sustained attention, this ability should be more developed in skilled martial arts athletes as a consequence of their training. Our hypothesis was that skilled athletes would show better performance and differences compared to novice athletes in both the negative and positive components of the MRCP related to high motor control.

### Methods

#### Participants

Twenty-two martial arts athletes from three different combat disciplines (judo, taekwondo and kung-fu) were recruited. The participants were divided into two groups: a) 12 skilled athletes (mean age = 25.5 years, *SD* = 10.6) with more than five years of practice and the highest degree in their discipline and b) 10 novice athletes (mean age = 24.3 years, *SD* = 9.7) with less than one year of practice and the lowest degree in their discipline. All participants were healthy and had normal or corrected-to-normal vision. All subjects included in the study had scores in the normal range (>90) on the Wechsler Intelligence Scale and an attention deficit and hyperactivity disorder (ADHD) score greater than −1.80 in the task of variables of attention (TOVA), which indicates normal attention (see Table 1).

**Table 1:** Group characteristics for chronological age, years of sport practice, ADHD score, Intelligence Quotient score and sport disciplines practiced.

Group	Age	Years of sport practice	ADHD score	IQ	Sport
Skilled *N* = 11	25±11	9±6	1.7±1.9	105±7.7	Judo = 5
					TKD = 4
					Kung-fu = 3
Novice *N* = 10	25±9	1±0	1.0±1.6	108±2.6	Judo = 3
					TKD = 2
					Kung-fu = 5
Skilled *vs.* Novice	NS	*p* = .01**	NS	NS	NS

NS = no significant differences between groups.

**p≤.01.

#### Ethics Statement

Participants were informed of their rights and provided written informed consent for participation in the study. Additionally, when participants enrolled in our study were minors, the written informed consent was obtained from their parents (mother or father) on behalf of them. This research was carried out ethically following the principles stated in the chapter five on ethics and medical research of the Declaration of Helsinki. Both this research and its informed consent were approved by the Ethics Committee of the Neurobiology Institute at the Universidad Nacional Autónoma de México.

#### Stimuli

Five different pointed arrows (2.95 cm width, 2.03 cm height; white on black background) were used. A sequence of arrows was presented in the center of a 17-inch VGA computer monitor at a viewing distance of 80 cm and visual angle of approximately 2.11×1.45°.

#### Continuous performance task

A sequence of 600 arrows divided into six blocks of 100 arrows was presented to each participant. The participants were instructed to respond to the target arrow (pointed right and downward) and not to respond when another arrow was shown. The target stimulus itself was presented in 20% of the trials and the non-target stimulus in 80%. The stimulus duration was 100 ms, and a variable interstimulus interval (1.2–1.5 s) was used.

#### Procedure

Participants were seated in a comfortable chair in a room with low light and instructed to respond by pressing a button with the right index finger as rapidly and accurately as possible when the target stimulus appeared during the task.

#### ERP recording

An electroencephalogram (EEG) was recorded with 32 Ag/Cl sintered surface electrodes mounted on an elastic cap (Electrocap) using NeuroScan SynAmps amplifiers (Compumedics NeuroScan) and Scan 4.5 software (Compumedics NeuroScan). The electrodes were referenced to linked earlobes. Electrooculograms were recorded from a supraorbital electrode and an electrode placed at the external canthus of the left eye. The EEG was digitized at a 500-Hz sampling rate and filtered using a band-pass filter set from 0.1 to 100 Hz. Electrode impedance was maintained below 5 kΩ.

#### Data analysis

Behavioral analysis was conducted using percentages of correct responses, which were transformed ARCSIN [SQRT(percentage/100)]. To analyze accuracy, a two-way ANOVA was performed with Group (skilled and novices) and Condition (target and non-target) as factors. A one-way ANOVA was also performed for reaction times.

The MRCP were computed offline using 2000-ms epochs from each subject in the target condition using motor response as the trigger. Each epoch consisted of 1500 ms preceding the motor response and 500 ms following the motor response. A baseline correction was performed using the initial 200 ms of the epoch. Epochs with voltage changes exceeding +100 µV were omitted from the final average. Vertical electro-oculogram artifacts were removed by applying an eye-movement correction algorithm [35]. Segments with artifacts and noisy electrical activity were rejected. The average number of EEG segments per condition and group was approximately equal (more than 60 trials).

Statistical analyses were performed on MRCP time windows selected by visual inspection according to previous literature [23]: The BP was divided into two components: early BP, measured as the mean amplitude between −800 and −400 ms, and the late BP, measured as the mean amplitude between −400 and −200 ms. The maximum negative amplitude was measured between −200 and −50 ms (N-115). To measure the positive activity around the response, the maximum positive amplitude between 0 and 100 ms (P50) was taken at electrodes from central, parietal and temporal areas. Additionally, the mean amplitude between −500 and −100 ms was considered in prefrontal areas where the pP positive activity was observed. The pN in the grand average was not clearly detected and was not analyzed.

For each component identified, an ANOVA statistical analysis was performed. Because the negative components were mainly observed in posterior areas prior to a motor response in skilled athletes and the same distribution was observed in a P50 component after motor response in novice athletes, only central, temporal and parietal electrodes were analyzed. Centro-Parietal (CP3, CP4, P3 and P4) and Temporo-Parietal (TP7–TP8 and T5–T6) electrodes were analyzed separately using Group (skilled and novice), Topography (central-parietal and parietal) and Laterality (left and right) as factors. To analyze electrodes on the centro-parietal midline (CPz and Pz), Group (skilled and novice) and Topography (centro-parietal and parietal) were used as factors. Finally, Group (skilled and novice) and Laterality (left, midline and right) were used as factors to analyze the prefrontal electrodes (Fp1, Fpz and Fp2). The Huynh-Feldt correction was applied to correct for the degrees of freedom. The minimum significant difference (MSD) criteria were used for post-hoc comparisons in the repeated-measures analyses. Only significant differences between groups and interactions that involved Group are reported.

### Results

The behavioral results showed no significant differences between groups in the Group×Condition interaction for the rate of correct responses (*F*(1,19) = .2, *p* = .7). Similarly, there were no differences between groups in response times (*F*(1,19) = .1, *p* = .7) (Table 2).

**Table 2:** Behavioral results for the CPT and c-CPT: percentages of target and non-target correct responses and response times for both the skilled and novice groups.

Task	Condition	Skilled	Novice
CPT	Target	*M* = 97.84±2.14	*M* = 97.59±1.52
	Non-target	*M* = 99.46±.43	*M* = 99.51±.29
	Response Time	*M* = 420 ms±51 ms	*M* = 428 ms±44 ms
c-CPT	Target	*M* = 95.55±4.73	*M* = 95±4.48
	Non-target	*M* = 99.47±.37	*M* = 99.69±.29
	Response Time	*M* = 366 ms±40 ms	*M* = 357 ms±51 ms

*M* = mean.

**Figure 1:**
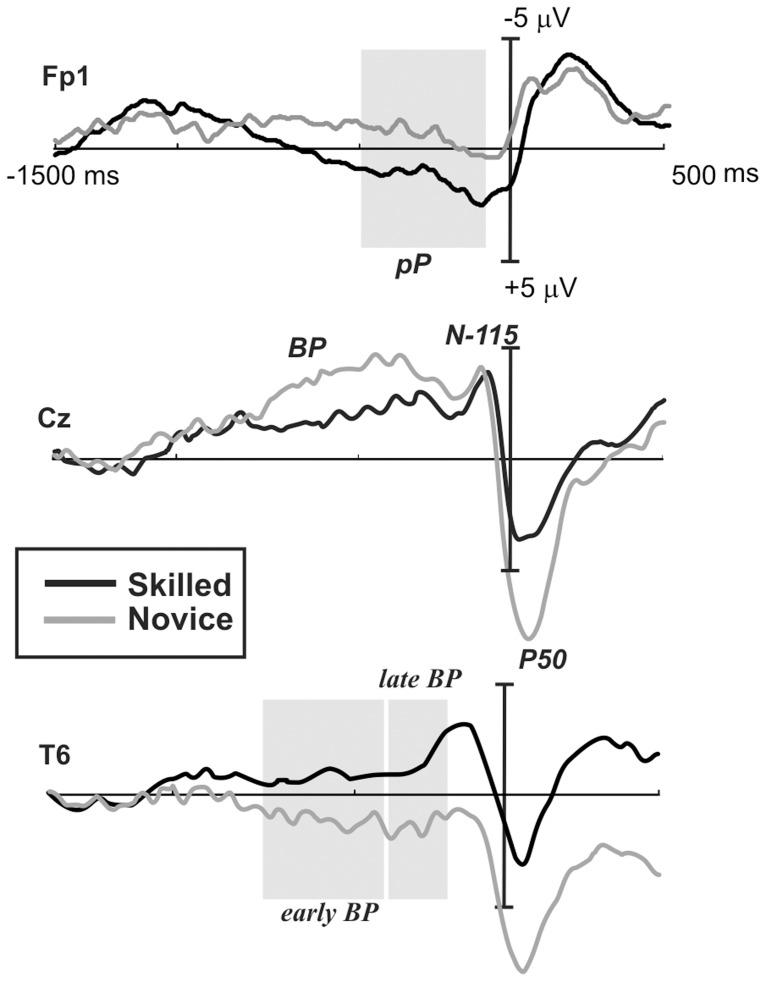
Grand average waveforms of MRCPs at the prefrontal, central and temporal electrodes for the CPT. Black lines indicate skilled athletes, and gray lines indicate novice athletes. Gray marks show windows where significant differences were found. Negativity is plotted up.

**Figure 2:**
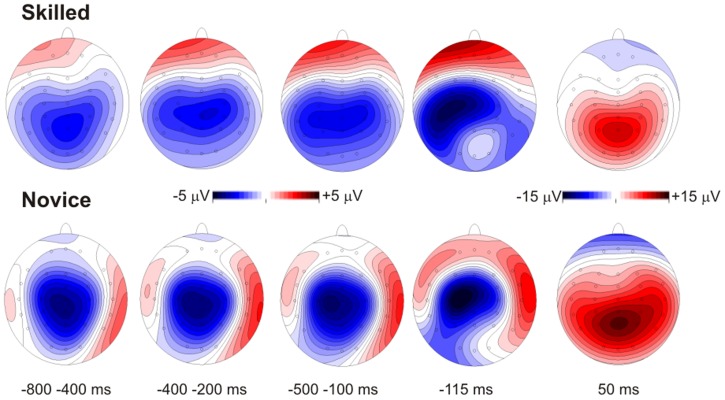
CPT amplitude maps for each time window analyzed where significant differences were found. Skilled athletes are shown above, and novice athletes are shown below.

Waveforms and topography maps are shown in Figures 1 and 2, respectively. The MRCPs initiated with a slow rising negativity (BP) approximately 1200 ms before the response with a radial distribution over the medial central areas in both groups. In novices, the BP also showed a bilateral positivity over temporal sites; in both groups, the BP reached the maximum negativity at approximately 115 ms before the response (N-115). In experts, a slow prefrontal positivity was also present concomitantly with the BP and was slightly larger over the hemisphere that was contralateral to the responding hand. These two activities terminated after the target stimulus onset, just before the response, and were substituted by a medial posterior parietal positivity corresponding to the stimulus-related P3 component, peaking approximately 50 ms after the response (P50). Statistical results are reported (Table 3).

**Table 3:** Significant differences between groups in the amplitude of the MRCP components in the CPT.

Component	Topography	Electrodes	Mean Difference	*F*_(1,20)_	*p*
Early BP	Temporo-parietal	All_(TP7,TP8,T5,T6)_	1.3 µV(1)	4.9	.03
Late BP	Temporo-parietal	TP8	2.5 µV(1)	4.8	.04
		T6	2.7 µV(1)		
pP	Prefrontal	Fp1	2.1 µV(1)	5.0	.03
Maximum Negative Amplitude	Temporo-parietal	TP7	4.2 µV(1)	5.7	.02
		TP8	3.7 µV(1)		
		T6	4.2 µV(1)		
P50	Temporo-parietal	All_(TP7,TP8,T5,T6)_	4.1 µV(2)	4.9	.03

(1) Amplitude was larger in skilled than novice athletes.

(2) Amplitude was larger in novice than skilled athletes.

#### Early BP (window from −800 to −400 ms)

Electrodes on temporo-parietal sites showed significant differences between groups *F*(1,20) = 4.9, *p* = .03, *η^2^* = .199. Skilled athletes showed larger amplitudes in this negative component than novice athletes (*MD* = 1.3 µV, *p* = .03). Centro-parietal and centro-parietal midline electrodes showed no significant differences between groups.

#### Late BP (window from −550 to −250 ms)

Analysis of temporo-parietal electrodes in this time window showed significant differences between groups *F*(1,20) = 4.8, *p* = .04, *η^2^* = .195. A significant Group×Topography×Laterality interaction was found *F*(1,20) = 5.4, *p* = .03, *epsilon* = 1, where skilled athletes showed larger amplitudes than novices athletes, principally in the TP8 (*MD* = 2.5 µV, *p* = .04) and T6 (*MD* = 2.7 µV, *p* = .005) electrodes. No significant differences were observed on centro-parietal and centro-parietal midline electrodes.

#### pP (window from −500 to −100 ms)

The results of the analysis on prefrontal electrodes showed significant differences between groups *F*(1,20) = 5.0, *p* = .03, *η^2^* = .200. Skilled athletes showed significantly larger Fp1 electrode amplitudes than did novices (*MD* = 2.1 µV, *p* = .01).

#### Maximum negative amplitude (∼−115 ms)

Analysis of temporo-parietal electrodes in this time window showed significant differences between groups *F*(1,20) = 5.7, *p* = .02, *η^2^* = .223. A significant Group×Topography×Laterality interaction was found *F*(1,20) = 8.2, *p* = .009, *epsilon* = 1, where skilled athletes showed larger amplitudes than novice athletes, principally in the TP7 (*MD* = 4.2 µV, *p* = .02), TP8 (*MD* = 3.7 µV, *p* = .04) and T6 (*MD* = 4.2 µV, *p* = .01) electrodes. No significant differences were observed on centro-parietal and centro-parietal midline electrodes.

#### P50

In this time window, differences in temporo-parietal electrodes between groups were found *F*(1,20) = 4.9, *p* = .03, *η^2^* = .197. The results showed larger amplitudes in novice athletes than in skilled athletes (*MD* = 4.1 µV, *p* = .03). No differences were found in centro-parietal and centro-parietal midline electrodes in this time window.

### Discussion

We hypothesized that skilled and novice athletes would show differences in performance and MRCP components during a sustained attention task. Our analysis showed no significant differences in behavioral performance between the groups. However, some MRCP components did differ. The negativity of the BP did not differ between groups. However, in novices, the BP was larger on the medial central electrodes, and it showed a less radial distribution, yielding bilateral positivity in the temporal areas that was not present in skilled athletes. Furthermore, skilled athletes group showed a strong prefrontal positivity concomitant with the BP and disappearing with response onset. Activity around the time of the response was dominated by the parietal P50, which coincided with the P3 component. This activity was much larger in novices.

Previous MRCP studies [24], [25], [26], [27], [28], [29], [30] have found a reduction in the BP component in skilled participants and have attributed it to smaller and more economic motor preparation in the supplementary motor areas of skilled individuals. The present study did not confirm this notion statistically; however, the results showed a similar trend for BP negativity. More interestingly, novices, but not skilled athletes, showed a bilateral positive counterpart of the BP over bilateral temporal areas indicating that their BP activity is produced by sources in the bilateral SMA with different orientations that converge during the negativity in medial central areas and diverge during the positivity in temporal areas. Concordantly with the aforementioned studies, these differences may be associated with the activation of a smaller portion of the SMA in experts (presumably around the SMA sulcus) than in novices, which could have also activated the lateral wall of the SMA.

The novel result here is the presence of a strong pP in skilled athletes compared to novice athletes. Differences between groups in prefrontal activity were found at Fp1, where skilled athletes showed larger amplitudes during a long period preceding motor response (from −600 to −150). Topography and laterality suggest that this prefrontal activity might be related to top-down supervision [36], which controls response selection during motor preparation. This prefrontal positivity has not been reported before using a CPT in athletes but was detected in recent MRCP studies using discriminative response tasks, such as go/no-go [31], [32], fatiguing leg action [37] and bimanual coordination, where it has been associated with awareness of the upcoming response execution. Because the CPT requires sustained and controlled attention, we propose that the prefrontal positivity could be related to higher executive control of motor responses in skilled athletes. Considering both simultaneous pP and BP activity, it is likely that skilled athletes are able to allocate two different processes, i.e., motor response preparation and motor control preparation, simultaneously. The presence of greater anticipation in prefrontal areas in the skilled group is consistent with the idea that skilled martial arts competitors must be able to cognitively anticipate actions and strategies [1].

During a CPT, it is necessary to maintain attention for long time periods because the stimulus is a potential target that may require a response; thus, the subject must be able to sustain and control attention and motor response preparation. This assumption supports our results, which reveal that skilled athletes have better mechanisms to process both of these demands. In terms of real sport practice, it is known that fighters use strategies that include focusing their attention on their own performance and the performance of their opponent and maintaining their attention to plan the speed and power of their motor responses [38], [39].

The positive peak found approximately 50 ms after a motor response coincided in timing and topography with the well-known stimulus-related P3 component associated with stimulus discrimination and motor response execution, but in this MRCP analysis, this peak should be considered a response-related P3. In this sense, considering that novice athletes showed greater amplitude in this component, we may assume that this group invests greater resources during this stage of the task. Skilled athletes, in contrast, invest more resources at earlier stages of a task to make the final result more efficient and accurate [1].

## Experiment 2

In the transient attention task, participants were instructed to respond only when the target stimulus was preceded by a signal stimulus. Many studies have shown that cued response preparation produces superior task performance compared to sustained response preparation [40], [41]. c-CPT tasks involve transient attention and transient response preparation because advanced response preparation is only called for if the signal stimulus is presented [34]. Because the ability to respond automatically in combat when specific movements are detected in the opponent is necessary for high-level athletes, they are required to hone their capacity for quickly detecting a target in order to conserve resources. We hypothesized that skilled athletes would show better performance compared to novice athletes. We also expected to see differences between groups in both the negative and positive components in the MRCP due to less motor control and more automatic motor responses in skilled athletes.. The aim of this experiment was to assess MRCP differences between skilled and novice martial arts athletes during a transient attention task. A cued continuous performance task (c-CPT) was used.

### Methods

#### Participants

The participants, characteristics and inclusion criteria were the same as for Experiment 1.

#### Stimuli

The same five different pointed arrows as in Experiment 1 were used. The instructions were changed to apply the same sequence to a transient attention task.

#### Cued continuous performance task

The participants were instructed to respond to the target arrow only if a specific arrow (warning stimulus) preceded it three stimuli before (go trial). Participants were presented with the following types of no-go trials: (i) target arrow not preceded by warning stimulus (false target); (ii) non-target arrow preceded by warning stimulus (false signal); and (iii) non-target arrow not preceded by warning stimulus (no cue trial). The warning-target sequence requiring the participant to make a motor response was presented in 10% of the trials. The warning stimulus itself was presented in 20% of the trials because it could precede the target or another stimulus. Thus, the occurrence of go and no-go trials was equiprobable.

#### Procedure

This experiment followed the same procedure and recording protocol as Experiment 1.

#### Data analysis

Statistical analyses was performed using MRCP time windows selected by visual inspection according to previous literature [23]: The early BP was measured as the mean amplitude between −900 and −600 ms, and the late BP was measured as the mean amplitude between −550 and −250 ms. To measure the positive activity around the response, the maximum amplitude between −50 and 50 ms (P10) was taken at electrodes from the central, parietal and temporal areas. Furthermore, the mean amplitude between −900 and −100 ms was also considered in prefrontal areas where the pP was observed. The pN in the grand average was not clearly detected.

For each value, an ANOVA statistical analysis was performed. Because a greater distribution of negativity was observed from the fronto-central to parietal areas in skilled athletes and the same effect was observed in the P10 component in novice athletes, fronto-central, central, temporal and parietal electrodes were analyzed. Central (FC3–FC4, C3–C4, CP3, CP4, P3 and P4) and Temporal (FT7–FT8, T3–T4, TP7–TP8 and T5–T6) electrodes were analyzed using Group (skilled and novice), Topography (frontal, central, centro-parietal and parietal) and Laterality (left and right) as factors. To analyze electrodes on the midline (FCz, Cz, CPz and Pz), Group (skilled and novice) and Topography (frontal, central, centro-parietal and parietal) were used as factors. Finally, Group (skilled and novice) and Laterality (left, midline and right) were used as factors to analyze the prefrontal electrodes (Fp1, Fpz and Fp2). The minimum significant difference (MSD) criteria were used for post-hoc comparisons in the repeated-measures analyses. Significant differences between groups were found and are reported. No significant interactions were found.

### Results

As in Experiment 1, the behavioral results showed no significant differences between groups in the Group×Condition interaction for the rate of correct responses (*F*(1,19) = .350, *p* = .561). Similarly, there were no differences between groups in response times (*F*(1,19) = .191, *p* = .668) (See Table 2).

**Figure 3:**
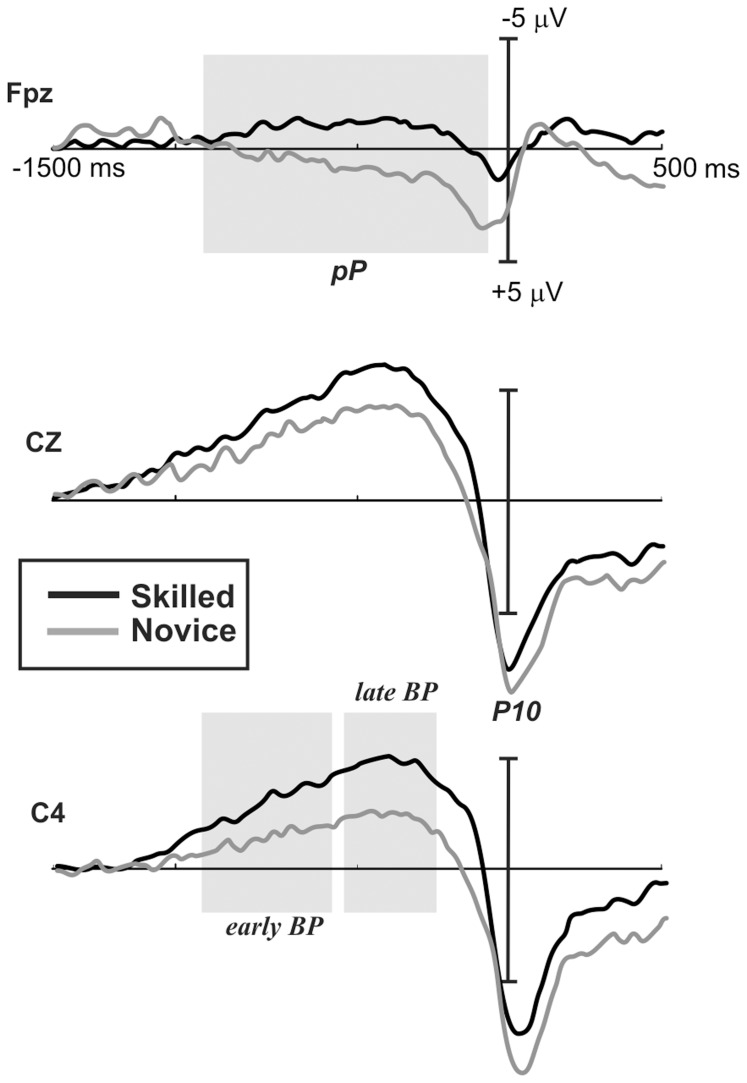
Grand average waveforms of MRCPs at the prefrontal and central electrodes for the c-CPT. Black lines indicate skilled athletes, and gray lines indicate novice athletes. Gray marks show windows where significant differences were found. Negativity is plotted up.

**Figure 4:**
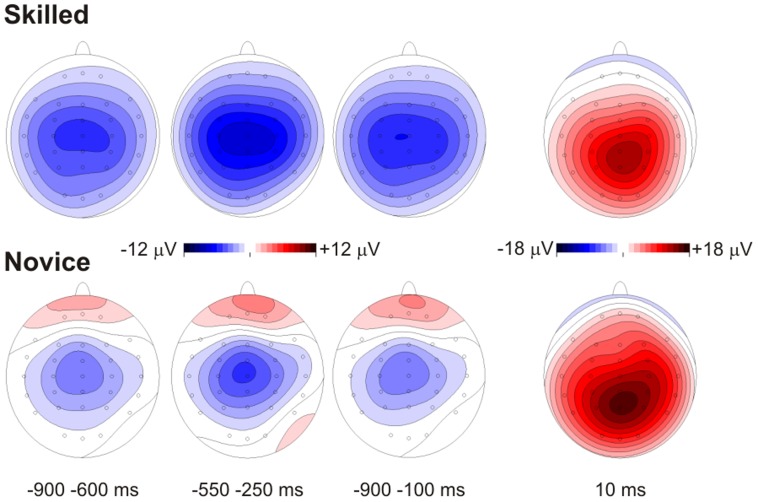
c-CPT amplitude maps for each time window analyzed where significant differences were found. Skilled athletes are shown above, and novice athletes are shown below.

Waveforms and topography maps are shown in Figures 3 and 4, respectively. The MRCPs initiated with a slow rising negativity (BP) approximately 1500 ms before the response, with a radial distribution over the medial central areas in both groups. In this task, the BP slope was steeper than in the task used in Experiment 1. In both groups, the BP reached maximum negativity at approximately 400 ms before the response. Concomitantly with the BP, novices also showed a slow pP with a radial distribution. These two activities finished after the target stimulus onset, just before the response, and were substituted by a medial posterior parietal positivity corresponding to the stimulus-related P3 component, peaking approximately 10 ms after the response (P10). Statistical analysis of the electrophysiological data showed significant group differences, indicating that skilled athletes showed a larger BP than novices, whereas the pP and the P10 were smaller. Statistical results are reported below (Table 4)

**Table 4:** Significant differences between groups in the amplitude of the MRCP components in the c-CPT.

Component	Topography	Mean Difference	*F*_(1,20)_	*p*
Early BP	Central	2.2 µV(1)	5.1	.03
	Temporal	1.7 µV(1)	5.7	.02
Late BP	Central	3.2 µV(1)	3.2	.02
	Temporal	2.6 µV(1)	2.6	.01
pP	Prefrontal	4.6 µV(2)	5.8	.02
P10	Temporal	3.3 µV(2)	5.5	.02

(1) Amplitude was larger in skilled than novice athletes.

(2) Amplitude was larger in novice than skilled athletes.

#### Early BP (window from −900 to −600 ms)

Electrodes on central sites showed significant differences between groups *F*(1,20) = 5.1, *p* = .03, *η^2^* = .203. Skilled athletes showed larger amplitudes in this negative component than novice athletes (*MD* = 2.2 µV, *p* = .03). When electrodes on temporal sites were analyzed, significant group differences were found *F*(1,20) = 5.7, *p* = .02, *η^2^* = .224, with larger amplitudes in the skilled group compared to the novice group (*MD* = 1.7 µV, *p* = .02). No significant differences were found when midline electrodes were analyzed.

#### Late BP (window from −550 to −250 ms)

Analysis of central electrodes in this time window showed significant differences between groups *F*(1,20) = 6.1, *p* = .02, *η^2^* = .234. Skilled athletes showed larger amplitudes than novices athletes (*MD* = 3.2 µV, *p* = .02). Similar results were found when temporal electrodes were analyzed *F*(1,20) = 7.6, *p* = .01, *η^2^* = .276, with skilled athletes consistently showing larger amplitudes than novices (*MD* = 2.6 µV, *p* = .01). No differences were found in midline electrodes.

#### pP (window from −900 to −100 ms)

The results of the analysis of prefrontal electrodes showed significant differences between groups *F*(1,20) = 4.1, *p* = .02, *η^2^* = .239. Novice athletes showed larger amplitudes in this positive component than skilled athletes (*MD* = 4.1 µV, *p* = .02).

#### P10

In this time window, differences in temporal electrodes between groups were found *F*(1,20) = 5.5, *p* = .02, *η^2^* = .216. The results showed larger amplitudes in novice athletes than in skilled athletes (*MD* = 3.3 µV, *p* = .02). No differences were found in central and midline electrodes in this time window.

### Discussion

Our hypothesis about this transient attention task was that differences in performance and MRCP components would be found between groups. Analysis of performance showed no differences, but differences between groups were observed in the MRCP components. The skilled group showed larger BP amplitudes at central electrodes than novices. This result contradicted previous studies where self-paced tasks were used. Furthermore, larger positive amplitudes were observed preceding motor response at prefrontal electrodes in novice athletes compared to skilled athletes, which is contrary to the findings of the first experiment. Additionally, a larger positive amplitude (P10) was found in novice athletes after a motor response.

The cue CPT is a task where automatic mechanisms of attention are required, i.e., less controlled attention and motor responses. Skilled athletes showed larger and more distributed negative BP activity starting 900 ms before the motor response, which likely relates to higher motor preparation and greater anticipation. On the other hand, novice athletes showed less negativity, which most likely relates to less motor preparation and anticipation. Additionally, bilateral prefrontal positive activity was found in novice but not in skilled athletes, suggesting higher cognitive control during the task. In general, group differences could be based on greater motor preparation in skilled athletes and more controlled cognitive processing in novice athletes. With these results in mind, we propose that skilled athletes show more automatic mechanisms of anticipatory and mental preparation and respond with fewer attentional resources.

## General Discussion

The aim of this study was to identify differences in motor-related cortical potential between skilled and novice martial arts athletes during sustained and transient attention tasks to study expertise effects. It has been proposed that sustained and transient tasks refer to two different types of cognitive processes [34]. Considering that sustained and transient attention are required during training and competition, we anticipated differences between groups due to the effects of expertise. There were no differences in behavioral performance in both tasks. One explanation could be that the stimuli were too easy to result in the expected differences. However, electrophysiological data can offer deeper analyses about the cognitive processes during task performance [42].

The MRCP results showed differences between groups, and these differences were distinct, depending on the type of task. Electrophysiological data in the CPT task indicated larger pP activity and greater posterior negativity distribution prior to a motor response in the skilled athletes, while novices showed significantly larger response-related P3 after a motor response in centro-parietal areas. We propose that skilled athletes are able to allocate two different but related processes simultaneously in the early stages of a task to control motor response preparation, which is consistent with CPT demand, where controlled attention and controlled motor responses are required. During the c-CPT task, novice athletes showed strong prefrontal positive activity before the motor response and a large response-related P3, whereas in skilled athletes, the prefrontal activity was absent. We propose that skilled athletes possess better cue facilitation, which permitted a major saving of resources and “automatic” or less controlled responses to relevant stimuli.

Two classes of attentional processing mechanisms have been proposed: automatic and controlled [43], [44]. Automatic processes are fast, inflexible and consume little attentional capacity. Conversely, controlled processes are slow, attentionally demanding and controlled by the participant's intentions. In our study, skilled athletes showed brain electrical activity that suggests controlled sustained attention. In the transient attention task, skilled athletes were able to process information more automatically after cue stimulus than were novices. Overall, skilled athletes appeared to process information more quickly and to spend less of their available attentional resources on transient attention processing than novice athletes.

## Conclusions

Our results indicated differences in visuo-motor processing between skilled and novice athletes during attentional tasks. Skilled athletes showed more controlled responses and greater anticipation in the early stages of the sustained attention task. Conversely, novice athletes showed more cognitive control and less motor preparation than skilled athletes during the transient attention task. Overall, there are differences in visuo-motor processing between groups, depending on the expertise level and requirements of the task. In general, the present data suggest that motor expertise enhances neural flexibility to allow better adaptation of cognitive control to a requested task.
